# Clinical approach to the male with delayed puberty

**DOI:** 10.20945/2359-4292-2025-0428

**Published:** 2025-11-24

**Authors:** Rodolfo A. Rey, Romina P. Grinspon, Sebastián Castro

**Affiliations:** 1 Centro de Investigaciones Endocrinológicas “Dr. César Bergadá” (CEDIE), CONICET - FEI - División de Endocrinología, Hospital de Niños Ricardo Gutiérrez, Buenos Aires, Argentina

**Keywords:** AMH, delayed puberty, FSH, inhibin B, testis

## Abstract

Disorders of pubertal onset and progression are a common cause for referral to paediatric endocrinologists, with delayed puberty in males being particularly frequent. Pubertal development depends on the hypothalamic-pituitary-testicular (HPT) axis, which is established during fetal life and undergoes distinct phases: fetal androgen production, postnatal “minipuberty”, and reactivation during adolescence. Key regulators include GnRH neurons, Sertoli and Leydig cells, and biomarkers such as AMH, inhibin B, testosterone and INSL3. Puberty is marked clinically by testicular enlargement beyond 4 mL, usually at a median age of 11.5 years. Delayed puberty is defined as absence of testicular enlargement by age 14. The most common cause is self-limited delayed puberty (SLDP), often familial and benign. Functional hypogonadotropic hypogonadism due to chronic illness, and permanent central hypogonadism (congenital or acquired), account for additional cases. Congenital hypogonadotropic hypogonadism (CHH), including Kallmann syndrome, is frequently genetic, with variants in genes such as *FGFR1, ANOS1* and *GNRHR*. Clinical assessment includes family history, growth patterns, and red flags such as micropenis, cryptorchidism or anosmia.

## INTRODUCTION

**Figure 1:**
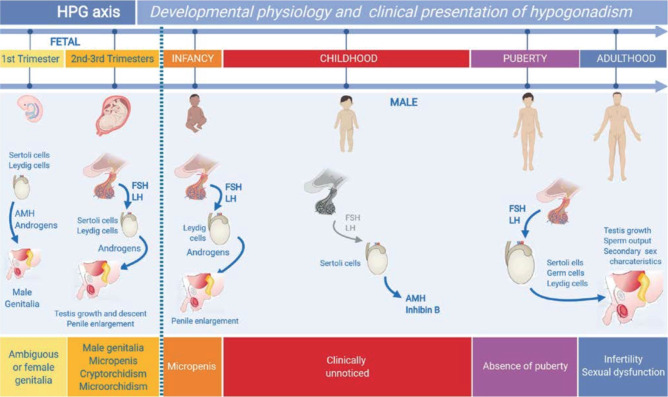
Ontogeny of the hypothalamic-pituitary-gonadal (HPG) axis in males, and its impact on clinical presentation of hypogonadism. In the first trimester of fetal life, the testis differentiates and secretes androgens and anti-Müllerian hormone (AMH), involved in genital sex differentiation, independently of pituitary gonadotropins. Hypogonadal states in this period lead to ambiguous or female genitalia. In the second and third trimesters, fetal FSH induces Sertoli cell proliferation and, consequently, testis enlargement, while LH regulates androgen secretion resulting in testicular descent and penile enlargement. Primary and central hypogonadisms result in micropenis, micro-orchidism and/or cryptorchidism. In infancy, gonadotropin and steroid secretion remain active for 3-6 months; hypogonadism prevents penile enlargement. During childhood. gonadotropins and androgens are normally low or undetectable; hypogonadism may go unnoticed unless AMH or inhibin B levels are assessed. During puberty, the axis is reactivated and results in pubertal maturation; hypogonadism leads to absent or incomplete sex development. Reprinted with permission from ref. (^66^).

Disorders affecting the onset or the progression of puberty are common reasons for referral to the paediatric endocrinologist. They are usually associated with a significant impact in the psychosocial sphere for both the patients and their families, and their management can be burdened with uncertainty. In this review, we will address the diagnosis and management of delayed puberty in males, based on the current knowledge of the physiology underlying the development of the hypothalamic-pituitary-testicular axis from fetal life through adulthood (**Figure 1**).

### Developmental physiology of the hypothalamic-pituitary-testicular axis From fetal life to prepuberty

Fetal differentiation of the testes is initially independent of pituitary gonadotropins (^1^). In the 7^th^ week of gestation, germ and Sertoli cells aggregate and form the seminiferous tubules; Sertoli cells secrete anti-Müllerian hormone (AMH), which induces the regression of the Müllerian ducts, *i.e.*, the anlagen of the uterus, Fallopian tubes and upper vagina, during weeks 8-10. In the 8^th^ week, Leydig cells differentiate in the interstitial tissue and start secreting androgens, involved in the virilization of internal and external genitalia during weeks 8-13, and insulin-like factor 3 (INSL3), which together with androgens induce testicular descent to the scrotum in the 2^nd^ and 3^rd^ trimesters of gestation. Androgen and INSL3 production are regulated by placental human chorionic gonadotropin (hCG) during the 1^st^ trimester of fetal life. The establishment of the hypothalamic-pituitary-gonadal axis occurs later, approximately during the 17^th^ fetal week (^2^). Then, LH regulates Leydig cell hormone production, and FSH induces immature Sertoli cell proliferation and upregulates AMH and inhibin B secretion (^3^). Specifically, AMH secretion is a typical feature of immature (prepubertal) Sertoli cell activity.

Unlike most neuron types of the central nervous system, the gonadotropin-releasing hormone (GnRH) neuron arises from the olfactory placode and migrates to its final position in the hypothalamus following the olfactory nerve. GnRH neuron specification, proliferation and migration are regulated by Anosmin 1 (encoded by *ANOS1*), Chromodomain Helicase DNABinding Protein 7 (*CHD7*), Dual-Specificity Phosphatase 6 (*DUSP6*), FEZ Family Zinc Finger protein 1 (*FEZF1*), Fibroblast Growth Factors 8 (*FGF8*) and 17 (*FGF17*) and their receptor FGFR1, Heparan Sulfate 6-O-Sulfotransferase 1 (*HS6ST1*), Interleukin 17 Receptor D (*IL17RD*), Neuron-Derived Neurotrophic Factor (*NDNF*), NMDA Receptor Synaptonuclear Signaling and Neuronal Migration Factor (*NSMF*, also known as *NELF*), Prokineticin 2 (*PROK2*) and its receptor PROKR2, SRY-Box 10 (*SOX10*) and WD Repeat-Containing Protein 11 (*WDR11*) (^4^). Once established in the hypothalamic nuclei, the GnRH neuron is regulated by Kisspeptin (*KISS1*) and its receptor KISS1R, Leptin (*LEP*) and its receptor LEPR and Tachykinin 3 (*TAC3*) and its receptor TACR3. Another set of factors are implicated in the morphogenesis of the hypothalamus and pituitary, including HESX Homeobox 1 (*HESX1*), Lim Homeobox gene 4 (*LHX4*), Nuclear Receptor subfamily 0, group B, member 1 (*NR0B1*, also known as DAX1), PROP paired-like homeobox 1 (*PROP1*), and the SOX family factors SOX2 and SOX3 (^5^).

Gonadotropin, testosterone and INSL3 levels remain high during the 2^nd^ trimester and decrease progressively in the 3^rd^ trimester of gestation (^6^), probably in response to placental oestrogens. At birth, all pituitary and testicular hormones are low (^7^-^9^). During the first postnatal week, gonadotropins levels increase (^8^) and remain high for 3 to 6 months in boys. LH induces an increase in testicular secretion of testosterone and INSL3 (^9^-^11^). This period of life has been called “minipuberty” given its similarity with puberty in terms of gonadotropin and androgen serum levels. Beyond the 6^th^ month, LH, testosterone and INSL3 levels decrease to very low or undetectable levels and remain so throughout childhood (**Figure 1**). Serum FSH, inhibin B and AMH also increase during the first week after birth. FSH and inhibin B decrease after “minipuberty”, but remain clearly detectable. For its part, the AMH remains high during childhood, reflecting the immature status of Sertoli cells (^12^).

**Figure 2:**
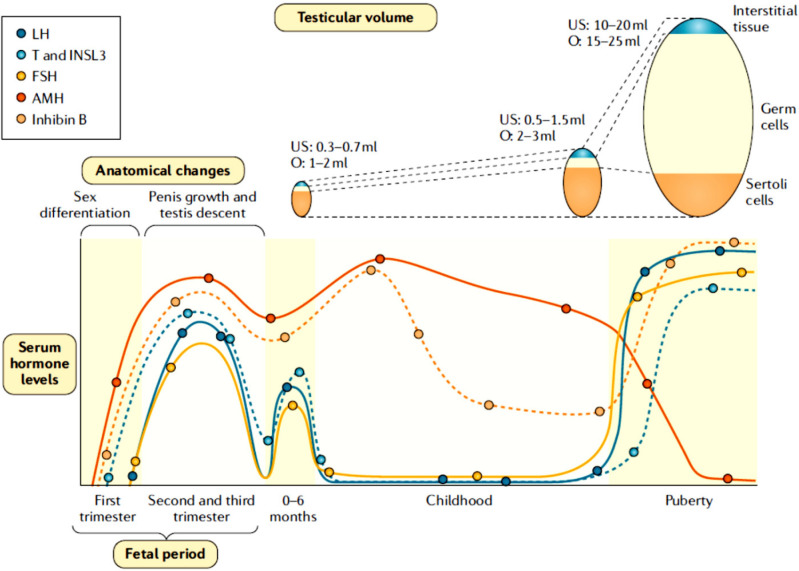
Serum hormone levels and changes in testicular size and genital features in males from fetal life through adulthood. Sertoli cells are the main component of the testes in fetal life and childhood, whereas germ cells are the major component during puberty and adulthood. INSL3: insulinlike factor 3; O: testicular volume measured by comparison to Prader’s orchidometer; US: testicular volume measured by ultrasonography. Reprinted with permission from ref. (^67^).

From a clinical standpoint, the size of the penis and testicular descent reflect the secretion and action of androgens in the 2^nd^ and 3^rd^ trimesters of gestation. On the other hand, testicular volume is a good indicator of FSH action, since the size of the testis mainly represents the number of Sertoli cells before puberty and Sertoli cell proliferation is FSH-dependent (^3^). Serum levels of LH, testosterone and INSL3 are good markers of the gonadotroph-Leydig cell axis from the 2^nd^ week of life and through “minipuberty”, *i.e.*, a maximum of 6 months. Conversely, FSH, inhibin B and AMH are biomarkers of the gonadotroph-Sertoli cell axis all through childhood (**Figure 2**). Despite the high intratesticular concentration of androgens during fetal life and “minipuberty”, Sertoli cells do not mature because they start to express the androgen receptor only by the end of the 1^st^ year of postnatal life (^13^,^14^). The germ cell population is limited to premeiotic spermatogonia.

#### Puberty

**Figure 3:**
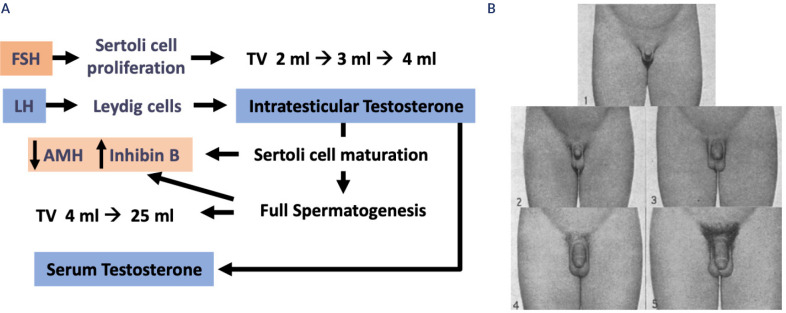
**A.** Testicular maturation during puberty. With the reactivation of the hypothalamic-gonadotroph axis, FSH increases immature Sertoli cell proliferation leading to testis enlargement from prepubertal (2 mL) to pubertal (4 mL) size. Concomitantly, LH induces Leydig cell differentiation and testosterone production. Initially, testosterone concentration increases within the testes and provoke Sertoli cell maturation. Mature Sertoli cells decrease anti-Müllerian hormone (AMH) secretion and become capable of supporting full spermatogenesis. The massive proliferation of germ cells results in a dramatic increase in testicular volume, up to 15-25 mL. FSH and germ cells induce an increase in Inhibin B. The elevation of serum testosterone to adult levels is a relatively late event, occurring between Tanner stages 3 and 5. **B.** Standards for genital rating during male puberty (stage 1: prepubertal; stage 5: adult), according to Marshall and Tanner. Reproduced with permission from ref. (^23^).

Puberty is the period characterised by the development of secondary sexual characteristics, the acquisition of fertility and accelerated growth and bone maturation leading to adult height. The underlying physiological process relies on a progressive increase in gonadotropin pulse amplitude and frequency (^15^). FSH induces the proliferation of immature Sertoli cells and drives the initial increase of testicular volume from ~2 to ~4 mL (**Figure 3A**). LH reactivates androgen production, leading initially to very high intratesticular concentrations that induce Sertoli cell maturation (^16^). Sertoli cell do not proliferate any longer, AMH production declines (^17^,^18^) and inhibin B secretion rises (^19^,^20^). Serum testosterone increases only later during pubertal maturation. Androgens are aromatised to oestrogens, which can result in physiological transient gynecomastia in more than half of normal boys during midto late puberty. Steroid hormones are responsible for the occurrence of peak height velocity and peak bone mass. INSL3 secretion also increases during puberty but becomes gonadotropin-independent in adult Leydig cells (^11^). Germ cells enter meiosis and undergo complete spermatogenesis, leading to sperm production (**Figure 1**) and to the characteristic progressive increase in testicular volume to ~15-25 mL (**Figure 2**) (^16^). FSH and germ cells upregulate inhibin B secretion, which in turn exerts negative feedback on pituitary FSH (^21^). In adolescents and adults, inhibin B levels are very informative, since they reflect the whole pubertal maturation process: FSH and testosterone action on Sertoli cells and the complete spermatogenic process.

In the male, the clinical milestone of the onset of puberty is testicular volume reaching 4 mL (**Figure 3B**), occurring at a median age of 11.5 years (^22^). It is associated with enlargement of the scrotum and a change in the texture as well as reddening of the scrotal skin in the initial stage of pubertal maturation, considered as Tanner genital stage 2 (^23^); during this stage testis volume increases up to ~6 mL (^24^). Subsequently, in Tanner genital stage 3, there is further growth of the testes (8-10 mL) and scrotum together with penile enlargement. In Tanner stage 4, the penis further enlarges with development of glans, together with growth of the testes (12-15 mL) and enlargement and darkening of the scrotal skin; maximal growth acceleration occurs. Finally, in genital stage 5, genitalia are adult in size and shape, with testes 15-25 mL.

### Delayed puberty

In boys, delayed puberty is defined by the absence of clinical signs of reactivation of the hypothalamicpituitary-testicular axis at an age at least 2 standard deviations later than that observed in the population mean. In practice, this means the failure to change from Tanner genital stage G1 to stage G2, characterised by enlargement of the testicular volume to reach 4 mL or testicular length to reach 25 mm, by the age of 14 years (^23^,^25^,^26^). Pubic hair development is not considered in the definition since it may reflect the activation of adrenal sex steroid secretion, *i.e.*, adrenarche (^27^).

The definition of pubertal delay is related to the timing of the onset of puberty. The fact that initial signs of puberty appear does not guarantee the completion of pubertal maturation. The pubertal tempo may be affected, resulting in an abnormally low progression and the adult stage is not reached within roughly 5 years after the initial signs of puberty (^23^); in some cases, pubertal maturation may come to a complete arrest. While pubertal delay may be transient, prolonged and arrested puberty usually reflect a morbid condition, as discussed below.

#### Aetiologies

The most frequent cause of the absence of pubertal signs by the age of 14 years in boys is self-limited delayed puberty (SLDP), also called constitutional delay of puberty. In these individuals, which represent 60%-80% of boys seeking medical attention for absence of pubertal signs (^26^,^28^-^30^), the onset of puberty usually occurs by the age of 18 years (**Table 1**). Another transient condition is functional central (or hypogonadotropic) hypogonadism, responsible for 10%-20% of the cases in males. It is associated with a wide variety of chronic or acute diseases, such as malnutrition, coeliac disease, cystic fibrosis, chronic kidney or liver disease, juvenile idiopathic arthritis and many others with chronic inflammation, Cushing syndrome, diabetes mellitus, *etc.* When the underlying disease is adequately managed, the function of the hypothalamic-pituitary-gonadal axis is reestablished, and puberty progresses. Conversely, permanent central (or hypogonadotropic) hypogonadism (HH), which represents 8%-10% of the boys with delayed puberty, can be acquired or congenital. Acquired forms may be due to tumours of the central nervous system and their radiotherapy, hypophysitis, or trauma affecting the hypothalamus and/ or pituitary, and are almost always associated with multiple pituitary hormone deficiencies. Congenital forms may also be associated with other pituitary hormone deficiencies, or they can present as isolated HH. Finally, primary (or hypergonadotropic) hypogonadism can be the underlying cause for the absence of puberty. This is rare in males (4%-5%) and is most often associated with anorchia (congenital or acquired), since primary testicular disorders, such as Klinefelter syndrome, longstanding cryptorchidism, orchitis or chemotherapy, usually affect germ and Sertoli cells more significantly than Leydig cells, which retain sufficient steroidogenic capacity to induce penile and scrotal growth.

**Table 1:** Aetiologies of pubertal delay in males

Aetiology	Relative frequency	Examples
Constitutional delay of growth and puberty (CDGP)	60%-70%	
Primary hypogonadism	2%-7%	Bilateral anorchia/Testicular regression syndrome Mild testicular dysgenesis Klinefelter syndrome (47,XXY and variants) Bilateral orchitis/chemotherapy
Congenital hypogonadotropic hypogonadism (genetic)	2%	Isolated CHH (with or without anosmia) Multiple pituitary hormone deficiency
Acquired central hypogonadism	4%-6%	Surgery of the sellar/suprasellar region Pituitary tumours Cranial trauma High dose cranial radiotherapy
Functional central hypogonadism	16%-20%	Systemic diseases (coeliac or inflammatory bowel disease, diabetes, malnourishment, hypothyroidism, etc), emotional stress

AMH: anti-Müllerian hormone; FSH: follicle-stimulating hormone; LH: luteinising hormone.

Congenital HH (CHH) most frequently has a genetic origin as a consequence of gene variants that impair early differentiation of the GnRH neurons at the olfactory placode or their migration to the hypothalamus, the regulation of GnRH secretion, or gonadotropin secretion in response to GnRH. Early embryonic defects involving GnRH differentiation or migration together with defects in the olfactory tract result in CHH with hyposmia or anosmia, known as Kallmann syndrome. Defects of GnRH synthesis, secretion or action result in normosmic HH (^4^,^31^-^34^). A recent systematic review of the phenotypegenotype correlation in males with CHH leading to absent or arrested puberty after the age of 18 years, with a metanalysis after reclassification of sequence variants following the recommendations of the American College of Medical Genetics and Genomics (ACMG) and the Association for Molecular Pathology (AMP) for the interpretation of the pathogenic potential of gene variants, identified 503 different disease-causing variants in 29 genes (**Table 2**) (^35^). These variants were associated with the absence of puberty (*i.e.*, complete forms of CHH) in 85.5% of the cases and with arrested puberty (*i.e.*, partial forms) in 14.5% of them. In males with complete CHH, variants in *FGFR1* and *ANOS1* accounted for 53.5% of all the disease-causing variants, whereas in patients with partial forms of CHH, variants in *FGFR1, NR0B1* and *GNRHR* were found in 70.3% of the cases.

**Table 2:** Characteristics of puberty, olfactory system and pituitary deficiencies in patients with disease-causing variants of congenital hypogonadotropic hypogonadism (CHH), according to the causal gene, in a systematic review and metaanalysis (^*^)

Gene	Index cases with disease-causing variants	Puberty			Olfactory disturbance			Abnormal olfactory bulb and tract			Other pituitary deficiencies		
		Absent	Arrested	NA	Yes	No	NA	Yes	No	NA	Yes	No	NA
	**n**		**n(%)**			**n(%)**			**n(%)**			**n(%)**	
*FGFR1*	153	133(86.9)	20(13.1)	0	103 (69.6)	45 (30.4)	5	58 (62.4)	35(37.6)	60	2(1.4)	141 (98.6)	10
*AN0S1*	101	95(94.1)	6(5.9)	0	94(94.9)	5(5.1)	2	41 (85.4)	7(14.6)	53	0(0.0)	82(100.0)	19
*NR0B1*	64	44(69.8)	19(30.2)	1	1 (2.7)	36 (97.3)	27	1 (6.7)	14(93.3)	49	1 (2.6)	37 (97.4)	26
*GNRHR*	47	34 (72.3)	13(27.7)	0	0(0.0)	47(100)	0	0 (0.0)	20(100.0)	27	0(0.0)	47(100.0	0
*CHD7*	24	20 (83.3)	4(16.7)	0	17(73.9)	7(26.1)	0	3 (27.3)	8 (72.7)	13	0(0.0)	22(100.0)	2
*TACR3*	17	15(88.2)	2(11.8)	0	0(0.0)	17(100)	0	0 (0.0)	9(100.0)	8	2(11.8)	15(88.2)	0
*KISS1R*	15	15(100.0)	0(0.0)	0	0(0.0)	14(100.0)	1	0(0.0)	12(100.0)	3	1(7.1)	14(92.9)	0
*SOX10*	12	9(75.0)	3 (25.0)	0	12(100.0)	0 (0.0)	0	7 (87.5)	1 (12.5)	4	0(0.0)	10(100.0)	2
*GNRH1*	11	11 (100.0)	0(0.0)	0	0(0.0)	11 (100.0)	0	0(0.0)	4(100.0)	7	0(0.0)	9(100.0)	2
*PR0K2*	8	6(75)	2 (25.0)	0	5(62.5)	3 (37.5)	0	4(66.7)	2 (33.3)	2	0(0.0)	7(100.0)	1
*PNPLA6*	8	6(75.0)	2 (25.0)	0	0(0.0)	3(100.0)	5	0(0.0)	4(100.0)	4	0(0.0)	4(100.0)	4
*S0X2*	7	6(85.7)	1 (14.3)	0	0(0.0)	3(100.0)	4	0(0.0)	3(100.0)	4	2 (28.6)	5(71.4)	0
*PR0KR2*	5	4(80.0)	1 (20.0)	0	3(^60^)	2(^40^)	0	1 (33.3)	2 (66.7)	2	0(0.0)	5(100.0)	0
*WDR11*	4	4(100.0)	0(0.0)	0	1 (33.3)	2 (66.7)	1	0 (0.0)	1 (100.0)	3	1 (50.0)	1 (50.0)	2
*FGF8*	4	4(100.0)	0(0.0)	0	2 (50.9)	2 (^50^,^9^)	0	0 (0.0)	3(75)	1	1 (25.0)	3 (75.0)	0
*P0LR3B*	3	3(100.0)	0(0.0)	0	0(0.0)	1 (100.0)	2	0(0.0)	2 (66.7)	1	1 (33.3)	2 (66.7)	0
*TAC3*	2	2(100.0)	0(0.0)	0	0(0.0)	2(100.0)	0	0 (0.0)	1 (100.0)	1	0(0.0)	2(100.0)	0
*FGF17*	2	2(100.0)	0(0.0)	0	2(100.0)	0 (0.0)	0	1 (100.0)	0(0.0)	1	0(0.0)	2(100.0)	0
*CPE*	2	2(100.0)	0(0.0)	0	0(0.0)	2(100.0)	0	0(0.0)	1 (100.0)	1	2(100.0)	0 (0.0)	0
*PLR3A*	2	2(100.0)	0(0.0)	0	0(0.0)	1 (100.0)	1	0 (0.0)	1 (100.0)	1	0(0.0)	1 (100.0)	0
*FEZF1*	2	2(100.0)	0(0.0)	0	2(100.0)	0(0.0)	0	2(100.0)	0(0.0)	0	0(0.0)	2(100.0)	0
*HESX1*	2	1 (50.0)	1 (50.0)	0	2(100.0)	0 (0.0)	0	0 (0.0)	0(0.0)	2	0(0.0)	2(100.0)	0
*RNF216*	2	2(100.0)	0(0.0)	0	0(0.0)	2(100.0)	0	0(0.0)	2(100.0)	0	0(0.0)	2(100.0)	0
*KISS1*	1	1 (100.0)	0(0.0)	0	0(0.0)	1 (100.0)	0	0 (0.0)	1 (100.0)	0	0(0.0)	1 (100.0)	0
*LEPR*	1	1 (100.0)	0(0.0)	0	0(0.0)	1 (100.0)	0	0(0.0)	1 (100.0)	0	0(0.0)	1 (100.0)	0
*LHB*	1	0(0.0)	1 (100.0)	0	0(0.0)	1 (100.0)	0	0 (0.0)	0(0.0)	1	0(0.0)	1 (100.0)	0
*NDNF*	1	1 (100.0)	0(0.0)	0	1 (100.0)	0(0.0)	0	1 (100.0)	0(0.0)	0	0(0.0)	1 (100.0)	0
*NHLH2*	1	1 (100.0)	0(0.0)	0	0(0.0)	1 (100.0)	0	0 (0.0)	0(0.0)	1	0(0.0)	0 (0.0)	1
*PR0P1*	1	1 (100.0)	0(0.0)	0	0(0.0)	1 (100.0)	0	0(0.0)	1 (100.0)	0	1 (100.0)	0 (0.0)	0
*SEMA3A*	1	1 (100.0)	0(0.0)	0	1 (100.0)	0(0.0)	0	1 (100.0)	0(0.0)	0	0(0.0)	1 (100.0)	0

Percentages are calculated based on available data. NA: data not available.

(^*^) Reprinted with permission from ref. (^35^).

Other conditions that can be associated with atypical forms of arrested puberty are X-chromosome polysomies, such as Klinefelter syndrome 47,XXY and its variants as well as in XX males (46,XX testicular DSD). These patients usually start and progress through puberty normally from an androgenic standpoint; however, mild hypoandrogenism may be observed in the latest stages of puberty. Furthermore, since there is meiotic failure with germ cell apoptosis and subsequent seminiferous tubule fibrosis, testicular volume arrests at 6-8 mL and then even regresses (^36^,^37^).

Self-limited delayed puberty shows high heritability. Although genome-wide association studies (GWAS) have identified several small-effect genes associated with pubertal timing (^38^-^40^), few causal genes have been identified in SLDP (^26^). A recent study on polygenic scores based on GWAS for timing of puberty in males and age at menarche in females has shown that common genetic variants associated with pubertal timing contribute to the genetics of SLDP, and that they are largely though not completely distinct from those associated with CHH (^41^). Another study in which burden tests analysed the frequency of rare variants in candidate genes for SLDP identified 14 high-impact and 7 moderate-impact variants in 19 candidate genes, with a potential role in pubertal delay (^42^).

#### Clinical assessment

As usual, a complete medical history and physical examination are the first steps in guiding the diagnosis in the cases of primary or acquired HH (^33^,^43^). Similarly, a deep phenotyping looking for extra-genital clinical manifestations could be helpful for the diagnosis of CHH. A recent systematic review (^35^) found associated manifestations in ~40% of patients with CHH carrying disease-causing gene variants. Particularly, adrenal insufficiency guided to variants in *NR0B1*, bimanual synkinesia or renal aplasia/dysplasia to defects in ANOS1, cleft lip and/or palate malformations and defects in dentition or hand/feet malformations were observed in association with variants in *FGFR1*, hearing defects guided to variants in *SOX10* and, when associated with facial dysmorphism and cardiovascular malformations, to variants in *CHD7* (Charge syndrome).

Conversely, the distinction between SLDP and isolated CHH may prove challenging. A family history of delayed puberty in parents, a gradual downward crossing of height centiles as compared to peers entering puberty, a delayed bone age and a delayed adrenarche are more characteristic of SLDP (^30^). Patients with SLDP present at slightly younger age, and are shorter and lighter than patients with HH (^44^). On the other hand, signs of deficient exposure to androgens in late fetal life and early infancy, such as micropenis and cryptorchidism, or of deficient exposure to FSH, leading to microorchidism, are considered “red flags” for the diagnosis of CHH (^28^,^33^,^44^). However, their absence should not rule out the diagnosis; indeed, they were present in less than 30% of males with *bona fide* gene variants of CHH (^35^). Cryptorchidism was more frequently associated with variants in *FGFR1, ANOS1, KISS1R, SOX10* and *GNRH1*, while micropenis was observed more frequently in patients with variants in *TACR3, KISS1R* or *GNRH1* (^35^).

#### Diagnosis

Elevated gonadotropin levels and and undetectable testosterone in the hormonal assessment rapidly points to primary hypogonadism. Functional hypogonadism may be ruled out using broad screening tests (*e.g.*, blood cell count, liver and kidney panels, blood chemistry, urinalysis, *etc.*); however, abnormal results are rarely found in the absence of clinical features or a history of a chronic illness (^43^). Imaging, especially magnetic resonance imaging (MRI), has a role in detecting central nervous system tumours, or defects in the olfactory tract guiding to the diagnosis of Kallmann syndrome.

**Figure 4:**
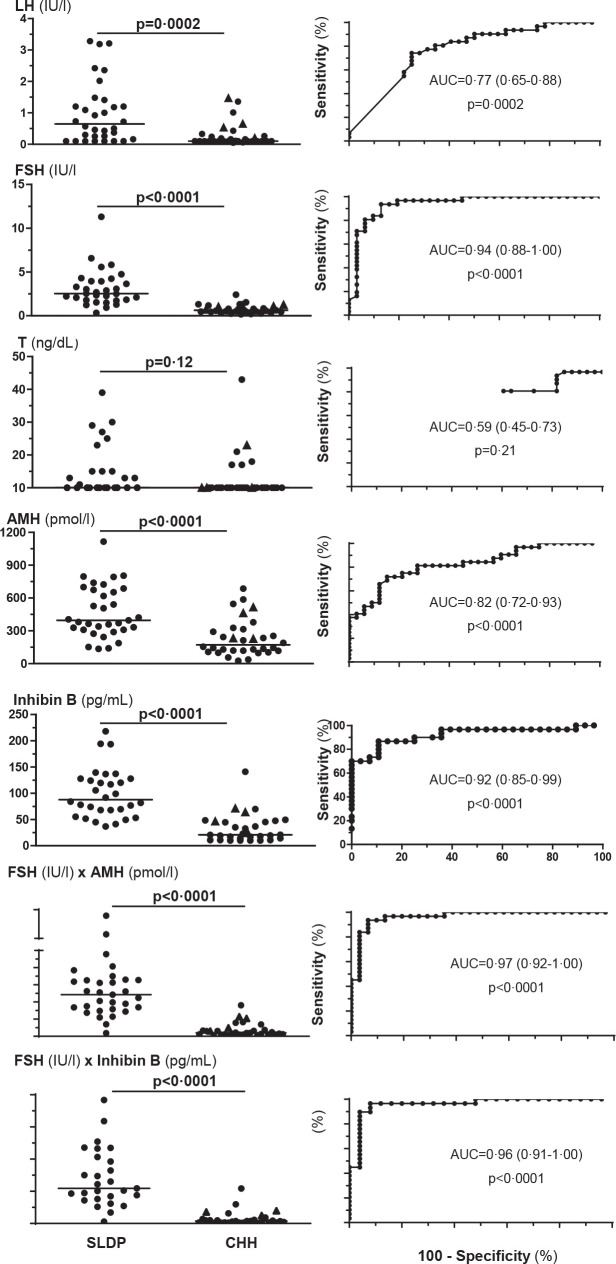
LH, testosterone (T), FSH, inhibin B and anti-Müllerian hormone (AMH) in the differential diagnosis between self-limited delayed puberty (SLDP) or congenital hypogonadotropic hypogonadism (CHH). Serum hormone levels at referral (main complaint: pubertal delay) in participants who were followed until a final diagnosis of SLDP or CHH was made: circles indicate complete form of CHH and triangles indicate partial form of CHH. ROC: receiver operating characteristic curves. AUC: area under the curve (95% confidence interval between parentheses). Reprinted with permission from ref. 48.

The use of laboratory tests for the differential diagnosis between SLDP and CHH, essentially based on the measurement of LH and/or testosterone under basal and stimulated conditions, has not been completely satisfactory for many years (^30^,^33^). Conversely, recent studies focusing on FSH and Sertoli cell biomarkers, such as AMH and inhibin B, have provided significant improvements in diagnostic accuracy (^45^-^48^). The use of the product FSH (IU/l)×inhibin B (ng/mL) < 92 or FSH (IU/l)×AMH (pmol/l) < 537 show high sensitivity (>93%), specificity (≥92%) and positive predictive value (>92%) for CHH in male patients aged ≥ 13 and < 18 years presenting with a testicular volume < 4 mL and no other sign of pubertal onset (**Figure 4**) (^48^). The efficacy of genetic testing has dramatically improved with the advent of high throughput techniques, such as next generation sequencing (NGS), allowing for massive parallel gene testing (^33^,^34^,^49^). The positive diagnostic yield of the underlying gene variant may exceed 50% in boys having undergone deep phenotyping (^4^,^34^,^48^,^50^,^51^).

#### Management

Boys with SLDP often present at the age of 12-13 years, with concerns about their relative short stature and lack of secondary sex characteristics as compared to peers. Reassurance of the patient and his parents and watchful waiting may be sufficient. However, when the condition causes psychosocial stress leading to negative social interactions and depression, pharmacological treatment may be necessary. In patients with a confirmed diagnosis of HH, medical intervention should not be delayed, provided that bone age is 11 years or more. Similarly, in adolescents with SLDP hormone treatment should be indicated by the age of 14 years in order to maximise height potential and peak bone mass and minimise psychosocial impact (^52^).

Testosterone is the most frequently used medication in boys with SLDP or HH (^28^,^33^,^52^,^53^), and the only one for males with anorchia or severe primary hypogonadism (^52^,^53^). Typical schemes start with testosterone enanthate or cypionate 50 mg IM every 4 weeks for 6 months, then increasing gradually by 50 mg every 6-12 months over 2 to 3 years until reaching the adult dose of 200-250 mg every 4 weeks. In patients showing testicular volume enlargement, especially during the initial stages of treatment with low doses, spontaneous onset of puberty is likely, and testosterone treatment can be discontinued. Nonetheless, monitoring is needed to ascertain that pubertal maturation is completed within 2-3 years. Other oral or transdermal testosterone formulations have been scarcely used to induce pubertal changes (^53^). Androgen treatment results in the development of secondary sex characteristics and increased growth and bone mineral density, leading to an improved psychological well-being (^53^,^54^). There are, however, certain drawbacks in the pharmacokinetics, since serum testosterone peaks to supraphysiological levels in the first week and falls to low levels in the fourth week (^55^). Side effects need to be monitored, especially by assessing total blood count, haematocrit, haemoglobin levels and liver function in order to identify polycythaemia or hypertransaminasaemia (^53^). Bone age advancement may also need to be monitored along with height velocity.

In individuals with SLDP, a 6-month treatment with oral letrozole 2.5 mg/day induced gonadotrophin secretion and testicular growth, together with accelerated height growth (^56^,^57^).

In patients with HH, replacement treatment should be centred on the administration of GnRH or FSH plus LH, if a physiology-based approach is applied. If gonadotrophins are used, the logical scheme should start with FSH in order to induce immature Sertoli cell proliferation and initial testis size enlargement, followed by the addition of LH or hCG (^58^,^59^). The latter results in testicular androgen secretion leading to Sertoli cell maturation and full spermatogenesis, together with the already described testosterone effects on secondary sex characteristics, growth and well-being. The increases in testicular volume and serum inhibin B are useful to assess the efficacy of treatment on spermatogenesis (^60^). Several treatment regimens exist. GnRH, which is not widely available, is administered with a mini-infusion pump delivering 25 ng/kg every 2 hours; dose titration may be needed to attain target serum testosterone (^61^). GnRH treatment seems to be more efficacious than gonadotrophins to induce spermatogenesis and increase testicular volume, with less oestrogenrelated secondary effects such as gynaecomastia (^62^). Gonadotrophin treatment regimens usually start with subcutaneous recombinant FSH 75-150 IU 2-3 times/week for 2-3 months, with dose adjustments up to 225 IU in order to ensure serum FSH levels of 4-6 IU/l for 2-6 months. Thereafter, FSH is combined with LH (75 IU/day) or hCG (500-2000 IU once or twice a week) with dose titration aiming for serum testosterone of 350 ng/dL for another 6 months, and LH or hCG alone for 6-12 months until testicular volume reaches 10-12 mL (^58^,^62^-^65^).

## FINAL REMARKS

Pubertal delay is a relatively frequent complaint in males. Primary hypogonadism is rarely the underlying cause; conversely, central hypogonadism may be difficult to diagnose timely based on clinical features and traditional biomarkers such as serum LH and testosterone levels. Some red flags, *e.g.*, a history of cryptorchidism, micropenis or microorchidism or smelling impairment, may guide the diagnosis. Serum levels of FSH and of Sertoli cell peptides, *i.e.*, AMH and inhibin B, have become excellent biomarkers to distinguish between SLDP and HH. When medical treatment is needed, testosterone administration is the standard of care, but replacement with GnRH or gonadotrophins have gained preference more recently. Whether the latter should be preferred over testosterone therapy in teenagers with HH remains unsolved. The use of GnRH or gonadotrophins appears as more “physiological”, with FSH preceding LH or hCG. However, the evidence-based answer should come from sufficiently powered controlled clinical trials in teenagers aged 12-14 years followed for long-term until adulthood.

## Data Availability

datasets related to this article will be available upon request to the corresponding author.
